# The impact of COVID-19 pandemic on AIDS-related mycoses and fungal neglected tropical diseases: Why should we worry?

**DOI:** 10.1371/journal.pntd.0009092

**Published:** 2021-02-09

**Authors:** Sanaz Nargesi, Felix Bongomin, Mohammad T. Hedayati

**Affiliations:** 1 Invasive Fungi Research Center, Communicable Diseases Institute, Mazandaran University of Medical Sciences, Sari, Iran; 2 Department of Medical Mycology, School of Medicine, Mazandaran University of Medical Sciences, Sari, Iran; 3 Department of Medical Microbiology and Immunology, Faculty of Medicine, Gulu University, Gulu, Uganda; 4 Global Action Fund for Fungal Infections (GAFFI), Geneva, Switzerland; Liverpool School of Tropical Medicine, UNITED KINGDOM

## Abstract

The World Health Organization (WHO) considers mycetoma, chromoblastomycosis, and paracoccidioidomycosis to be fungal neglected tropical diseases (FNTDs). Depending on climatic, cultural, and economic contexts, these diseases have a similar geographical distribution as many other diseases, particularly tuberculosis (TB) and malaria, but are often less targeted by the national and many international healthcare systems. Another subgroup of fungal infections, such as candidiasis, cryptococcosis, pneumocystosis, histoplasmosis, and to a lesser extent, aspergillosis, are known as AIDS-related mycoses. Although antiretroviral therapy (ART) has been able to decrease the mortality rate of these diseases, particularly cryptococcosis, the disproportionately low distribution of funds to their diagnosis and treatment remains an obstacle in saving and improving the lives of patients affected. A new wave of viral diseases dubbed the Coronavirus Disease 2019 (COVID-19) hit the world at the end of 2019. Due to progressive symptoms and high mortality rates of COVID-19 compared to fungal infections, particularly the FNTDs, funding is currently allocated predominantly for diagnostic and therapeutic research on COVID-19. As a result, advances in FNTDs and AIDS-related mycosis care are considerably reduced. This paper explores the association between COVID-19, FNTDs, and AIDS-related mycoses with a predictive perspective.

## Introduction

There are thousands of known fungal species throughout the environment. Given the growing number of existing fungal species, more than 300 species have been reported as human pathogens, causing a wide range of diseases ranging from asymptomatic to mild mucocutaneous diseases to severe and life-threatening disseminated infections. Among the different fungal genera, *Aspergillus*, *Candida*, *Cryptococcus*, and *Pneumocystis* are the most common opportunistic fungi that cause the majority of global morbidity and mortality attributable to fungal infections [1]. These infections are often seen in patients with factors such as HIV/AIDS, prolonged corticosteroid therapy, hematological malignancies, organ transplantation, and long-term hospitalization [2].

On the other hand, fungal neglected tropical diseases (FNTDs) are endemic in poor communities living in the tropical and subtropical regions all over the world. In the recent past, control of fungal infections, especially the FNTDs, has been targeted by national governments and international organizations, including the World Health Organization (WHO). The development of better diagnostic tools for the diagnosis, treatment, and management of fungal infections are however deemed far less important than that of other mycobacterial (tuberculosis, TB), parasitic (malaria), and viral infections (HIV/AIDS). Even though the morbidity and mortality inflicted by the FNTDs in affected communities are comparable to those of HIV/AIDS, TB, or malaria, fungal diseases continue to be a neglected subject, receiving less attention in terms of epidemiologic surveillance, diagnostic and therapeutic advances, and human resource at all levels.

To complicate this further, the ongoing Coronavirus Disease 2019 (COVID-19) pandemic caused by the novel Severe Acute Respiratory Syndrome Coronavirus 2 (SARS-CoV-2), which was first reported in 2019 in Wuhan, China, has paralyzed and continues to derange all sectors [3]. COVID-19 has infected approximately 35 million, and more than a million people have died in less than a year and hence is unarguably the most important challenge of the 21st century, with currently no known effective treatment or vaccine [4]. The question is, how will this emergent challenge impact on the consideration of healthcare systems for established health issues including FNTDs and AIDS-related fungal infections, which are often prevalent in poor regions of the world?

## What comes about when both FNTDs and COVID-19 run in parallel?

**Fig 1:**
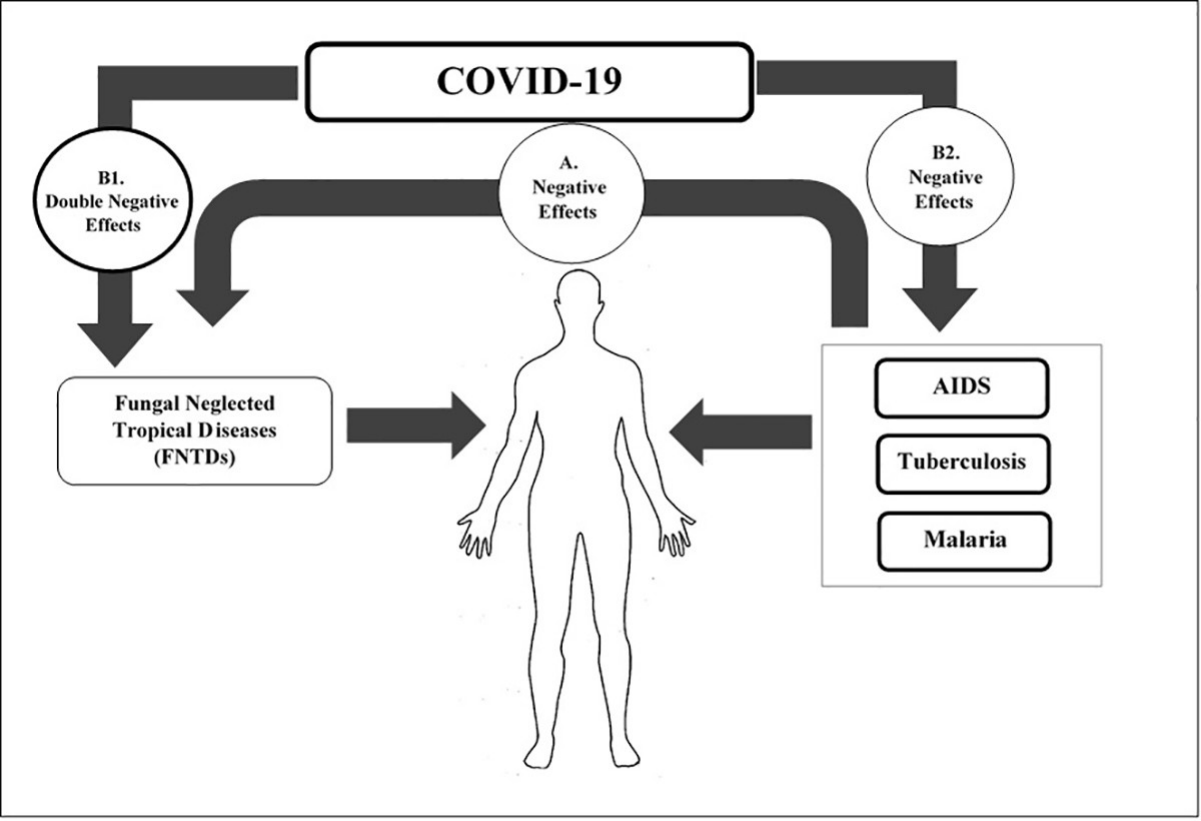
Proposed negative effects. (A) Negative effects of AIDS, malaria, and TB on FNTDs: In endemic areas, people are at risk for AIDS, TB, malaria, and FNTDs. But for reasons such as lower diagnostic focus and inadequate allocated budgets and longer common periods, FNTDs have not received the attention they need, despite their high prevalence. (B1) Negative effects of COVID-19 on FNTDs: On the other hand, with the global prevalence of COVID-19, with the high potential for morbidity and mortality, all the focus of healthcare is on that. Therefore, FNTDs will have other negative effects due to not receiving diagnostic and control requirements, and because they have previously been negatively affected by other diseases such as AIDS, TB, etc., the addition of COVID-19 will cause them to have **double negative effects**. So with the current situation, it is not unreasonable to call them **“Re-Neglected Fungal Tropical Diseases.”** (B2) Negative effects of COVID-19 on AIDS, malaria, and TB. COVID-19, Coronavirus Disease 2019; FNTD, fungal neglected tropical disease; TB, tuberculosis.

FNTDs are often reported in individuals with a low socioeconomic status. Even though clear-cut schemes are prepended to screen and mitigate this subset of diseases, the sharing out of higher funds to diagnosis and treatment of diseases, such as HIV/AIDS, TB, and malaria (typically overlapped with NTDs in geographic distribution), made these topics as a major concern. Unlike the high prevalence of FNTDs, the fairly long latency period and maybe even the existence of a number of misconceptions among the people of the endemic areas has meant this group of diseases are assumed an unusual occurrence. FNTDs continue to be heavily neglected, in spite of being potentially perilous within the healthcare field. Among different fungal diseases, mycetoma, sporotrichosis, and some other deep fungal diseases are on the list of FNTDs endorsed by WHO [5]. However, the Global Action Fund for Fungal Infections (GAFFI) has suggested paracoccidioidomycosis and fungal keratitis to the showcase of FNTDs group (https://www.gaffi.org/). Fungal NTDs have been adversely influenced by other infections in different ways. In a recent review on previous reports of financial support for research against various infectious diseases, fungal infection research had the least allocation [6]. Research in cryptococcal meningitis (CM) is the only fairly well funded of the myriad of other fungal infections, since it is the world’s fourth most deadly infectious disease. However, there is almost no funding allocation for the FNTDs, including those listed by WHO mentioned above [6,7]. With the advent of COVID-19, a new scenario can potentially be imagined for FNTDs. As COVID-19 is a global challenge, the coexisting of FNTDs and COVID-19 has at least 2 negative fallouts: (A) In the case of FNTDs, clinical manifestations typically take a long time to turn up, while the occurrence of COVID-19 is very fast and in some cases life-threatening. Therefore, in individuals with both types of infections, treatment of COVID-19 will unquestionably be a primary concern. (B) As more research is geared toward the complete understanding of the pathophysiology, treatment, and control of COVID-19, a lot of money shall be spent on combating this disease in all countries of the world. So the financial plan will be much more limited for the FNTDs especially in low-resource settings where these diseases are one of the main health problems (https://www.who.int/docs/default-source/health-financing/how-to-budget-for-covid-19-english.pdf?sfvrsn=356a8077_1). These 2 assumptions are among the most fundamental glitches foreseen when FNTDs and COVID-19 simultaneously occur. Finally, diseases such as TB and malaria have had a far-reaching negative effect on FNTDs over the past years [8]. COVID-19 will predictably not improve the situation for FNTDs, and the double negative effects outline in Fig 1 is anticipated. Accordingly, we suggest a new title for the FNTDs named “Re-Neglected Fungal Tropical Diseases” in the COVID-19 era (Fig 1). It may not be far-fetched that special teams will be developed in the near future to manage the simultaneously occurrence of both FNTDs and COVID-19.

## COVID-19 and AIDS-related mycoses

Opportunistic fungal infections (OFIs) due to *Aspergillus*, *Candida*, *Cryptococcus*, *Pneumocystis*, and *Histoplasma* species are the most frequent AIDS-related mycoses [1,2]. On the one hand, *Pneumocystis* pneumonia (PCP) due to *Pneumocystis jiroveci* is a major cause of OFI in AIDS patients with a mortality rate of up to 40% [2]. CM is the leading cause of meningitis among patients with advanced HIV particularly in sub-Saharan Africa [2]. Although antiretroviral therapy (ART) has reduced the incidence of CM, based on the economic background of the various countries in the world, the survival rate differs significantly [2]. Overall, the annual fungal burden of CM was 223,100, and the number of patients who died was estimated to be 181,100 [9]. Histoplasmosis associated with AIDS in endemic areas has a very high mortality rate (up to 70%) in some cases [2]. However, systemic candidiasis has only been occasionally reported, and esophagitis due to *Candida* species is one of the most prevalent infections in AIDS patients, occurring in up to 20% of those who are ART naïve [10]. Several cases of various clinical forms of aspergillosis with a 3.5 per 1,000 of 35,252 HIV–positive adults have been diagnosed in HIV/AIDS, per year [11]. Invasive pulmonary aspergillosis (IPA) is a well-known complication, mainly in immunocompromised and/or neutropenic patients, and carries a considerable mortality. IPA has been well described also among patients experiencing severe influenza admitted to intensive care units, where IPA prevalence rates of 16% to 23% have been reported [12]. It has been reported that IPA can be a notable complication in COVID-19, especially in cases with acute respiratory distress syndrome (ARDS) [12]. Indeed, reports about IPA in severe COVID-19 cases have started to emerge reporting prevalence of aspergillosis COVID-19 superinfections at 26.3% to 33% among patients presenting with ARDS [12], and therefore within the range of that observed for severe influenza [12]. Overall, mortality rates between 33% and 60% have been reported for COVID-19-associated IPA [12]. Accurate diagnosis of IPA in COVID-19 patients remains the main challenge. Besides infections caused by well-known fungal agents, we are currently confronted with a considerable number of emerging fungal agents in individuals with AIDS. *Candida auris* is an emerging fungal species and has been isolated from AIDS patients in most parts of the world, including North America, the Far East, Europe, and Africa (https://www.cdc.gov/fungal/candida-auris). *C*. *auris* has also been reported to cause candidemia in ICU patients with COVID-19 [13]. *Talaromyces marneffei* infection is also reported from HIV-infected individuals in Southeast Asia [2]. It is worth noting that a newer species known as *Talaromyces atroroseus*, which has a different pattern of antifungal susceptibility to *T*. *marneffei*, was reported in AIDS patients [14]. *Emergomyces africanus*, *Emergomyces orientalis*, and *Emergomyces pasteurianus* are 3 dimorphic fungal species that cause emergomycosis. *E*. *africanus* has been isolated from AIDS patients mainly in South Africa [15]. It is now well established that emergomycosis is very deadly; however, the influence of COVID-19 on this fungal infection remains unclear.

Fungal superinfection in combination with AIDS or COVID-19 can have several adverse implications. Suppressed cellular immunity promotes infection of most opportunistic fungal diseases associated with AIDS and COVID-19. In fact, each of these issues can be seen as the vertex of a triangle. In short, the simultaneous occurrence of weakened immune system, AIDS-related mycoses, and COVID-19 can form the vertices of a **“danger triangle**.**”**

In conclusion, the COVID-19 pandemic is a continually evolving state that will jeopardize progress toward the management of the FNTDs in many ways. As we are trying to control the COVID-19 crisis, it should always be remembered that the FNTDs do exist and continue to inflict suffering upon poor communities across the world. Furthermore, diagnostics and therapeutic advances should be geared toward early diagnosis and management of fungal diseases in the COVID-19 settings.
